# Quantifying the added value of new biomarkers: how and how not

**DOI:** 10.1186/s41512-018-0037-2

**Published:** 2018-07-11

**Authors:** Nancy R. Cook

**Affiliations:** Division of Preventive Medicine, Brigham and Women’s Hospital, Harvard Medical School, 900 Commonwealth Ave. East, Boston, MA 02215 USA

**Keywords:** Biomarkers, Model fit, Calibration, Reclassification, Clinical utility

## Abstract

Over the past few decades, interest in biomarkers to enhance predictive modeling has soared. Methodology for evaluating these has also been an active area of research. There are now several performance measures available for quantifying the added value of biomarkers. This commentary provides an overview of methods currently used to evaluate new biomarkers, describes their strengths and limitations, and offers some suggestions on their use.

During the past few decades, there has been an explosion of work on the use of biomarkers in predictive modeling and whether it is useful to include these when evaluating risk of clinical events. As new biologic mechanisms have been discovered, genetic markers evolved, and new assays developed, questions about the usefulness of new markers for clinical prediction have been debated. In cardiology, several strong risk factors for cardiovascular disease, namely cholesterol levels, blood pressure, smoking, and diabetes, have been well-known for decades [1] and have been incorporated into clinical practice. They have also been included in predictive models for cardiovascular disease, primarily developed in the Framingham Heart Study [2]. Since then, many new markers with more modest effects have been discovered as new biologic pathways have been unearthed. In fields which have less powerful predictors to date, development and addition of predictive markers may be even more important.

As interest in biomarkers has soared, so has the methodology used to evaluate their utility. There are now several performance measures available for quantifying the added value of biomarkers (Table 1), several of which have been proposed in the last decade. This commentary provides an overview of methods currently used to evaluate new biomarkers, describes their strengths and limitations, and offers some suggestions on their use.

**Table 1:** Summary of performance measures for quantifying added value

Measure	Advantages	Disadvantages
Likelihood-based measures	Reflects probability of obtaining the observed data	Based on assumed model
Likelihood ratio (LR), change in AIC or BIC	The LR test is the uniformly most powerful test for nested models. The AIC and BIC can be used to assess non-nested models.	While powerful, statistical association or model improvement may not be of clinical importance.
Discrimination	Assesses separation of cases and non-cases	Only one component of model fit
Difference in ROC curves, AUC, *c*-statistic	Assesses discrimination between those with and without outcome of interest across the whole range of a continuous predictor or score. Useful for classification	Based on ranks only. Does not assess calibration. Differences may not be of clinical importance.
Clinical risk reclassification	Examines difference in assigning to clinically important risk strata	Strata should be pre-defined. Loses information if strata are not clinically important
Reclassification calibration statistic	Assesses calibration within cross-classified risk strata	A test for each model is needed
Categorical NRI	Can assess changes in important risk strata. Cases and non-cases can be considered separately	Depends on the number of categories and cut points used
NRI(*p*)	Nice statistical properties. Does not vary by event rate in the data	May not be clinically relevant
Conditional NRI	Indicates improvement within clinically important risk subgroups	Biased in its crude form, and a correction based on the full data is needed.
Category-free measures	Does not require cut points	May lose clinical intuition
Brier score	Proper scoring rule	May be difficult to interpret; the maximum value depends on incidence of the outcome.
NRI(0)	Continuous, does not depend on categories	Based on ranks only. Measure of association rather than model improvement. Behavior may be erratic if the new predictor is not normally distributed.
IDI	Nice statistical properties. Related to the difference in model *R*^2^	Depends on event rate. Values are low and may be difficult to interpret.
Decision analytics	Estimates clinical impact of using model	Not a direct estimate of model fit or improvement. Need reasonable estimates of decision thresholds
Decision curve	Displays the net benefit across a range of thresholds	Does not compare model improvement directly but clinical consequences of using the models for treatment decisions
Cost-benefit analysis	Compares costs and benefits of one models or treatment strategy vs. another	Need detailed estimates of costs and benefits of misclassification, including further diagnostic workup and treatments

## Likelihood functions

A fundamental construct for much of statistical modeling is the likelihood function. This reflects the probability, or “likelihood,” of obtaining the observed data under the assumed model, including the selected variables and their associated parameters [3]. As more variables are added and the model fits the data better, the probability of obtaining the data that are actually observed improves. Much of statistical theory is based on this function. Thus, the primordial criterion of whether new variables, including biomarkers, can add to or improve a model is whether and by how much the likelihood increases. When the models are nested, we can test improvement with a likelihood ratio test, though other related tests, such as a Wald test, are sometimes used. For nonparametric models or machine learning tools, other loss functions are often used, such as cross-entropy or deviance, which are functions of the log likelihood for binary outcomes [4].

Other likelihood-based measures do not directly perform a test of significance, but apply a penalty for added variables, such as the Akaike Information Criterion (AIC) or Bayes Information Criterion (BIC). These are particularly valuable when non-nested models are used. While the original AIC applied a penalty of 2 degrees of freedom per variable, a generalized version uses an arbitrary penalty. The BIC, sometimes called the Schwarz criterion, applies a usually larger penalty of ln(*N*) where *N* is the number of observations. It thus favors more parsimonious models than the AIC, making it harder for a new biomarker to be judged to have added value.

The first criterion for assessing the addition of a new marker to a model should be the test of association, preferably based on a likelihood ratio test if models are nested. Several authors have argued that one test is all you need to assess new markers [5, 6]. Indeed, if a new marker cannot improve the likelihood or reduce entropy, then it is unlikely to have any clinical impact. Other tests can become redundant or even biased when the null hypothesis of no effect is true. Demler et al. [7] showed, based on *U*-statistics, that other tests of improvement, including the difference in area under the ROC curve, as well as the NRI and IDI described below, are degenerate and non-normal when comparing nested models under the null hypothesis.

While likelihood-based or deviance measures or testing can indicate significant associations or better fit for a model, this does not necessarily translate into clinical significance. The improvement may be small, may be limited to a few, or may otherwise fail to influence clinical decisions.

## ROC curves

Key components for evaluating medical tests are the sensitivity and specificity. These measures of discrimination, or separation of cases and non-cases, can be more helpful in determining how well a test can classify patients into those who truly have the disease or not. The sensitivity and specificity can be summarized over a range of cut points for a continuous predictor using the receiver operating characteristic (ROC) curve and the area under the curve (AUC). The AUC, also known as the *c*-statistic, can be interpreted as the probability that a case and non-case pair is correctly ordered by a model or rule [8]. By comparing two AUCs, one may compare the performance of diagnostic algorithms [9]. A similar measure, the c (for concordance) index, has been developed for survival data [10, 11]. This index, however, is dependent on the censoring distribution. Another concordance index, Gönen and Heller’s *k* [12], has been developed specifically for the Cox regression model and is robust to censoring [13]. Uno et al. [14] also introduced a modified c-statistic that does not depend on the study-specific censoring distribution.

For many years the *c*-statistic was the primary metric for evaluating diagnostic tests or prognostic models. While it bears some relation to measures of association estimated through statistical models, such as an odds ratio from a logistic regression [15], the odds ratio does not describe a marker’s ability to classify individuals. In fact, many promising biomarkers for cardiovascular disease, while having strong associations with CVD, fail to change the AUC to a meaningful extent, leading to pessimism about biomarker research [16, 17].

The ROC curve compares predictions across the whole range of risks by showing how well the model can rank predicted probabilities. It can be useful for classification, where discriminating between those with and without disease is of most interest. The ROC curve directly assesses discrimination and is not affected by calibration, or how well the predicted risks match those observed. When comparing models, changes in ranks may or may not be as important in patient care as changes in the levels of absolute risk. It would be possible, for example, for a new model to make small changes among many participants at low risk without changing risk estimates in those at higher and more clinically important risk. Conversely, and likely more common, big changes in risk among those at clinically important levels may not be acknowledged when looking solely at ranks [18].

## Risk reclassification

Clinical risk reclassification is an alternative that considers important risk strata and how well models classify individuals into these groups [19]. It focuses on risk strata that may be clinically important in prioritizing needs and targeting treatment decisions. A risk reclassification table can show how many people would be moved to new strata based on a different model or after adding a new biomarker. It is most useful when strata have been pre-defined and are useful clinically either for treatment decisions or for further attention and follow-up.

Besides the number changing strata, though, it is important to assess whether the changes are accurate. One way to do this is through the reclassification calibration statistic [20]. This compares the observed and predicted risks from each model separately within cells of the reclassification table, leading to two chi-square statistics, similar to Hosmer-Lemeshow tests, one for the old and one for the new model. This assesses the calibration within clinical meaningful categories and especially in those that are changed. For assessing calibration across a wide range of risk, it is usually helpful to use at least four risk strata so that estimated risk is more homogeneous within strata. If strata are not pre-defined, then cut points at p/2, p, and 2p, where p is the incidence or prevalence of disease, may be useful [20]. If a new biomarker adds to a model, then the old model should demonstrate a lack of fit in the new strata, which is improved with the new model. A drawback of this method is the need for two tests. There is also no measure of overall effect, although discordant observed and expected rates within the cross-classified risk strata can indicate where fit may be lacking.

Several measures and various extensions have been developed based on the risk reclassification table. The net reclassification improvement (NRI) [21, 22], which has become popular in the medical literature, computes the proportions moving up or down in risk strata in cases and non-cases separately. The overall NRI is the sum of improvement in each set. If cut points are based on clinical criteria, the NRI can indicate whether there are changes in risk stratification that can affect decisions regarding clinical care. The value of the NRI can, however, be affected by the number and size of strata, so these should be pre-specified [23]. A weighted version, in which weights are assigned based on the number of categories moved, has been described with particular reference to the three-category NRI [24]. Those estimates are generally similar to the original version unless there is substantial movement across two or more categories. Note that the components of the NRI should always be reported separately for those with and without events, so that these can be appropriately interpreted and reweighted if desired [22].

More recently, a two-category version of the NRI, with a cutoff at the event rate in the data has been described [25]. This NRI(p) has some nice statistical properties as it is equivalent to the net reclassification from the null model, to the difference in the maximum Youden index, and to the difference in the maximum standardized net benefit (described below). It is a proper measure of global discrimination measure that serves as a measure of distance between the distributions of risk between events and non-events. A problem is that it may not be clinically relevant [26, 27]. If a model is calibrated in the large, so that the average predicted risk equals the observed event rate, the cutoff will be at the mean predicted risk. If the risk estimates are normally distributed, then this will classify about half at high risk and half at low risk, but this may not be of clinical importance for treatment decisions. If the event rate is low, the distribution of risk estimates may be highly skewed and the mean will be higher than the median. In this case, fewer than half would be classified as high risk, but it may still be far too many to be clinically meaningful. For example, in the Women’s Health Study [28], the average risk for CVD over 10 years was approximately 3%, and about 28% would be classified as high risk. This threshold is lower than the 7.5% used in current recommendations for statin therapy [29]. Ideally, cost-benefit considerations should be used to obtain the optimal thresholds to translate these measures to clinical importance.

Clinicians are sometimes interested in identifying a subgroup for whom further diagnostic workup may be particularly warranted. For example, if an individual has a risk score in the intermediate range, further biomarkers or tests could be run such as recommended by the ACC/AHA guidelines for cardiovascular disease [29]. Using reclassification methods to look at change in the intermediate risk group only, though, can lead to bias. Even when there is no improvement or only random changes in category, this conditional or intermediate NRI can be large and statistically significant, with a large type I error [30]. A correction for this bias is available, but estimating model improvement in this subgroup nonetheless depends on having data across the full spectrum of risk [31].

## Category-free methods

A category-free version of the NRI (often denoted as NRI > 0) has also been described [32]. This determines whether risk increases to any extent for cases under a new model compared to the old or reference model, and similarly whether risk decreases to any degree for non-cases. While this does not require pre-specified categories and does not lose information due to categorization, such small up or down movement may also not be relevant clinically. The NRI > 0 is based on ranks only and is similar to a nonparametric test of association for a new biomarker. The size can also be distorted if the new variable or biomarker is not normally distributed [33]. Pencina et al. [34] have found that this measure tracks with the odds ratio and behaves as a test of association rather than of model improvement when the new predictor is normally distributed. When the predictor is not normally distributed, its behavior can be erratic.

Another measure that does not use categories but integrates the NRI over all levels of risk is the integrated discrimination improvement (IDI) [21]. This is equal to the difference in the Yates slope for one model vs. another and is equal to the difference in Brier scores scaled by their maximum possible value, or *p*(1 − *p*) where *p* is the event rate in the population. It is asymptotically equivalent to the proportion of the explained variation, a generalization of *R*^2^ [35, 36], and is thus related to the likelihood or change in entropy. The IDI as well as the NRI, however, can be strongly affected by the event rate [26]. As for *R*^2^ measures for binary or survival models, the values of the IDI are typically low and difficult to interpret. The relative IDI, which divides by the Yates slope in the reference model, is an alternative [37].

Note that while closed formulas are available for the standard errors of the NRI and IDI, bootstrapping is preferred for confidence interval construction [38]. In addition, while originally developed for binary outcomes, all versions of the NRI [32] as well as the IDI [39] and reclassification calibration statistic [40] are available for survival outcomes.

Hilden [41, 42] has demonstrated that unlike the Brier score, neither the IDI nor the NRI, whether continuous or categorical, is a proper scoring rule, though the IDI is asymptotically equivalent to the rescaled Brier score and is thus asymptotically proper [43]. This means that the NRI and IDI could indicate improvement for biomarkers with no added value. This often occurs when the models are not well-calibrated, leading to incorrect probabilities. The NRI and IDI are measures intended to assess discrimination, while the Brier score assesses both calibration and discrimination. Leening et al. [44] suggest that when considering the value of an individual added biomarker in an external validation population, it may be necessary to recalibrate the candidate models to provide a direct assessment of the individual biomarker’s contribution to discrimination. When comparing two different prediction models, however, calibration and discrimination are both essential components of model performance and need to be evaluated carefully.

## Choice of risk thresholds

The influence of thresholds is important to understand in evaluating models and their improvement. While theoretically attractive, measures based on the full range of risks, such as the *c*-statistic or NRI > 0 may not be relevant if they are based on ranks only. For reclassification calibration, it may be more useful to keep the number of risk categories somewhat larger so that the estimated risk is more homogeneous within strata. For the NRI, however, the choice of cut points is critical since this measure can vary widely depending on the number of strata [33]. The thresholds should ideally reflect important clinical risk strata which may be useful in determining treatment strategies, so that changes in these have clinical implications. Ideally, the cut points should be based on cost-effectiveness analysis, using the relevant costs of false positives and false negatives, both in terms of ethical and financial costs. The optimal threshold is a function of these. If the model is well-calibrated, the optimal threshold is 1/(1 + *t*) where the tradeoff *t* is the ratio of the net cost of misclassifying a case and the net cost of misclassifying a non-case.

In cardiovascular disease, a threshold of 0.075 has been suggested as a starting point for statin therapy [45]. This corresponds to a tradeoff of 12 to 1, implying that it is 12 times worse to misclassify a case than a non-case. Whether this is appropriate depends on treatment options, how well those alter risk, and their costs and side effects. If the overall event rate is used as the threshold, this is the point where sensitivity equals specificity, which may be optimal or not depending on the application. The NRI(*p*) based on this event rate may classify too many individuals as high risk, particularly if the outcome is rare. The low prevalence of rare events offers a unique challenge and also affects the performance of the various measures [26]. If the prevalence is as low as 1/2000, the cut point based on this would be 0.0005, equivalent to a cost tradeoff of 2000 to 1. Modelers need to work with clinicians to determine whether these values are appropriate. Models would also need to be very precise to discriminate between individuals at these very low levels of risk.

A full cost-benefit analysis can be useful in making treatment decisions or choosing to pursue additional testing, but this is complex. This is particularly helpful when clinical trade-offs exist for a treatment, with both benefits and harms. Even if the decision is whether to do further diagnostic follow-up [46], there may be harms related to unnecessary testing, both in terms of financial costs and in terms of unnecessary procedures or incidental findings. The net benefit, which is a proper measure, compares the benefits and risks of decisions, weighting by their relative harms or tradeoff. Choosing a specific threshold or tradeoff can be avoided by examining a decision curve, which plots the net benefit of a treatment or further diagnostic workup across a range of reasonable thresholds [47]. The decision curve is a decision-analytic tool to illustrate and compare the net benefit from treating all patients, treating none, or following a predictive model. It can also be used to compare the clinical consequences of two models across a range of thresholds and to determine whether clinical decisions based on these would lead to more good than harm [48]. Miscalibration also reduces estimates of net benefit; using models that severely over- or under-estimate risk may even lead to harm, particularly at thresholds further from the observed event rate [49].

A standardized version of the net benefit divides by the prevalence of the outcome, reaching a theoretical maximum value of 1, which improves interpretation [50]. This has also been called the relative utility [46]. As noted above, this is linked to the NRI(*p*) which is a difference in the maximum relative utilities across all thresholds for two models, occurring at the event rate [25]. This may mask clinically relevant thresholds, although it should not be far from the maximum achievable difference in relative utility [51]. While the NRI(*p*) provides a single number summary, it may be preferable to examine the whole relative utility curve to determine appropriate thresholds.

## Importance of validation

For all measures of model improvement, it is very important to compute unbiased estimates. When the testing or estimation is done in the data used for model development, models will generally be overfit and estimates of improvement optimistic. Adding more variables always leads to an apparent increase in performance. This is particularly true when the models are not pre-specified and variable selection or model-fitting is optimized. This may be ameliorated using penalized regression, such as lasso or ridge regression, which place penalties on added variables [4]. When adding a single new biomarker, the extent of over-fitting may be small, particularly in a large data set. In general, however, at least internal validation should always be performed. When model fitting is simple, internal validation through resampling, such as with bootstrapping or X-fold cross-validation, is preferable. If model fitting is more complex or more difficult to replicate, then dividing the data into test and training samples, ideally taking an average over multiple splits, is appropriate if sample size is sufficient. External validation in other data, settings, or with different patient samples should be done before any model should be implemented clinically to examine generalizability, including both reproducibility and transportability [52].

## Conclusion

There is no one ideal method to evaluate the added value of new biomarkers. Several methods evaluate different dimensions of performance and should be considered, depending on the need and stage of model development (Table 2). First and foremost, the new marker should be associated with the outcome and preferably have biologic validity. Unlike most epidemiologic analyses, though, a causal relation is not required for a marker to be a good predictor. The primary means of examining association is through likelihood-based measures, though likelihood ratio testing is applicable only to nested models. The IDI provides a nonparametric estimate and test of association, though its levels may be difficult to interpret.

**Table 2:** Recommendations

1. Test for model improvement using a likelihood-based or similar test. 1a. The IDI may be used as a nonparametric test or measure of effect if the models are well-calibrated. 1b. The NRI > 0 may be misleading, especially if a new marker is not normally distributed.	
2. Assess overall calibration and discrimination of each model. 2a. Plot observed and expected risk in categories or continuously with a smoother and compute the calibration intercept and slope. 2b. Compute the ROC curve and AUC or *c*-statistic if discrimination across the whole range of risk is of interest.	
3. If relevant risk strata are available, compute the risk reclassification table with clinical cut points or the overall prevalence, if relevant. 3a. Assess improvement in calibration within cross-classified categories. 3b. Assess improvement in discrimination through the categorical NRI.	
4. If relevant, consider bias-corrected conditional NRI to enhance screening of individuals at intermediate risk.	
5. If pre-specified risk strata are not available, consider cost tradeoffs to develop appropriate cut points.	
6. Consider decision analysis to assess the net benefit of using models for treatment decisions. 6a. Decision curves can be used to compare treatment strategies across a wide range of thresholds. 6b. Conduct full cost-effectiveness analysis if appropriate and estimates available.	
7. Validate *all* measures or tests of improvement in data not used to fit or select models. 7a. Internal validation, using bootstrapping, *X*-fold cross-validation, or (ideally multiple) split samples is required. 7b. External validation is preferable, particularly prior to clinical use.	

Basic requirements for model fit are that a new model is well-calibrated, with discriminant ability at least as good as previous standards, provided that costs are similar. To directly compare models, differences in ranks, such as assessed through ROC curves, may be useful, but may hide important differences in absolute risk. Several versions of risk reclassification methods have been proposed as alternatives. When clinical categories or risk strata make sense, then the reclassification calibration statistic or the categorical NRI can assess whether the new model fits best in these strata. When sensible strata do not exist, then category-free measures, such as the IDI, may be useful. While the event-rate NRI can apply generally and has nice statistical properties, it may or may not be clinically applicable.

Finally, to help determine if a model may be used to assign particular treatments or to decide on further testing, cost tradeoffs can be used to develop risk strata. A full decision analysis can be used to evaluate the net benefit of including new biomarkers. Alternatively, the decision curve can compare strategies across a wide range of thresholds.

Model development is not complete, however, without validation, at least internally, but preferably externally as well. Models intended to be applied to real patients need to be reproducible and generalizable to other applicable populations and settings.
